# *In situ* single photon confocal imaging of cardiomyocyte T-tubule system from Langendorff-perfused hearts

**DOI:** 10.3389/fphys.2015.00134

**Published:** 2015-05-06

**Authors:** Biyi Chen, Caimei Zhang, Ang Guo, Long-Sheng Song

**Affiliations:** Division of Cardiovascular Medicine, Department of Internal Medicine, Francois M. Abboud Cardiovascular Research Center, Carver College of Medicine, University of IowaIowa City, IA, USA

**Keywords:** T-tubules, *in situ* imaging, confocal microscopy, myocardium, E–C coupling, calcium

## Abstract

Transverse tubules (T-tubules) are orderly invaginations of the sarcolemma in mammalian cardiomyocytes. The integrity of T-tubule architecture is critical for cardiac excitation–contraction coupling function. T-tubule remodeling is recognized as a key player in cardiac dysfunction. Early studies on T-tubule structure were based on electron microscopy, which uncovered important information about the T-tubule architecture. The advent of fluorescent membrane probes allowed the application of confocal microscopy to investigations of T-tubule structure. Studies have now been extended beyond single cardiomyocytes to examine the T-tubule network in intact hearts through *in situ* confocal imaging of Langendorff-perfused hearts. This technique has allowed visualization of T-tubule organization in their natural habitat, avoiding the damage induced by isolation of cardiomyocytes. Additionally, it is possible to obtain T-tubule images in different subepicardial regions in a single intact heart. We review how this state-of-the-art imaging technique has provided important mechanistic insights into maturation of T-tubules in developing hearts and defined the role of T-tubule remodeling in development and progression of heart failure.

## Introduction

In the heart, the highly organized transverse (T)-tubule network provides the ultrastructural basis for rapid electrical excitation, initiation and synchronous triggering of Ca^2+^ release from the sarcoplasmic reticulum (SR), all of which are essential for coordinated myocyte contraction (Brette and Orchard, 2003; Ibrahim et al., 2011; Guo et al., 2013). Studies using electron microscopy and high resolution optical microscopy provided ample evidence that T-tubules are orderly extensions of the surface sarcolemma that run transversely along Z-lines at a regular spacing of ~2 μm with a diameter of 200–400 nm (Fawcett and McNutt, 1969; Kostin et al., 1998; Soeller and Cannell, 1999; Savio-Galimberti et al., 2008; Wagner et al., 2012). The concept of the “cardiac dyad” was first established based on the tight association of T-tubules with the terminal cisternae of the SR, also visualized by electron microscopy (Nelson and Benson, 1963; Rostgaard and Behnke, 1965; Fawcett and McNutt, 1969). We now understand that precise communication between voltage-gated L-type Ca^2+^ channels (LTCCs) located mainly on the T-tubule membrane and Ca^2+^ release channels/ryanodine receptor channels (RyRs) on the SR is essential for normal excitation–contraction (E–C) coupling (Stern, 1992; Sun et al., 1995; Wang et al., 2001; Guo et al., 2013). In the present review, we will discuss how the T-tubule *in situ* confocal imaging technique has emerged as a new approach to ask questions regarding ultrastructural changes in intact hearts.

## Methods for *in situ* confocal imaging of T-tubules

### Issues with imaging dissociated living myocytes

For many years, electron microscopy was the only method available to visualize T-tubule ultrastructure. Many previous studies using electron microscopy have provided valuable information and ultrastructural view of T-tubules and its organization within the cardiomyocytes (Nelson and Benson, 1963; Rostgaard and Behnke, 1965; Simpson, 1965; Ayettey and Navaratnam, 1978; Di Maio et al., 2007; Hayashi et al., 2009). However, electron microscopy studies have several caveats, including the complex and time-consuming steps of fixation, dehydration, embedding, sectioning, etc. In 1999, two-photon molecular excitation microscopy and digital image-processing methods were applied to examine T-tubules in living cardiomyocytes (Soeller and Cannell, 1999). This approach overcame the technical limitations of electron microscopy and allowed visualization of the exquisite complexity of the T-tubule system in live cells. Since then, many groups reported the use of two photon or single photon confocal microscopy to study T-tubule structure in living cardiomyocytes (He et al., 2001; Louch et al., 2006; Song et al., 2006; Heinzel et al., 2008; Dibb et al., 2009; Lyon et al., 2009; Ohler et al., 2009; Stolen et al., 2009; Ibrahim et al., 2010; Wagner et al., 2012). However, all of these studies were limited to single isolated myocytes. Enzymatic dissociation of myocytes may impair the T-tubule membrane of healthy cells. Also, those myocytes with severely damaged T-tubule membranes may be more fragile due to enzymatic digestion, mechanical stirring, and changes to cellular processes such as Ca^2+^ unloading and reloading during myocyte isolation process. These factors intrinsic to the isolation of cardiomyocytes are an obstacle to identifying subtle changes in T-tubule membrane structure in disease. In addition to using living isolated cardiomyocytes, fixed myocardium tissue sections were also used for T-tubule studies (Kaprielian et al., 2000; Crossman et al., 2011; Richards et al., 2011; Wu et al., 2012). This is particularly useful for imaging T-tubule structure of myocytes from large mammals and heart samples from human. In this approach, wheat germ agglutinin (WGA) conjugated to an Alexa Fluor is commonly used to label plasma membrane including T-tubules (Chazotte, 2011). Because of the advantage of WGA being able to label T-tubules in fixed cells, WGA labeling has been used as a marker of T-tubules in combination with immunolabeling of other E–C coupling or T-tubule associated proteins (e.g., LTCCs, RyRs, Na^+^/Ca^2+^ exchanger, carveolin-3, etc.) for simultaneous examination of the organization and spatial relationship between T-tubules and key E–C coupling proteins in health and diseased hearts (Jayasinghe et al., 2009; Crossman et al., 2011; Sachse et al., 2012).

### Technical details of *in situ* imaging

#### Langendorff perfusion setting

**Figure 1:**
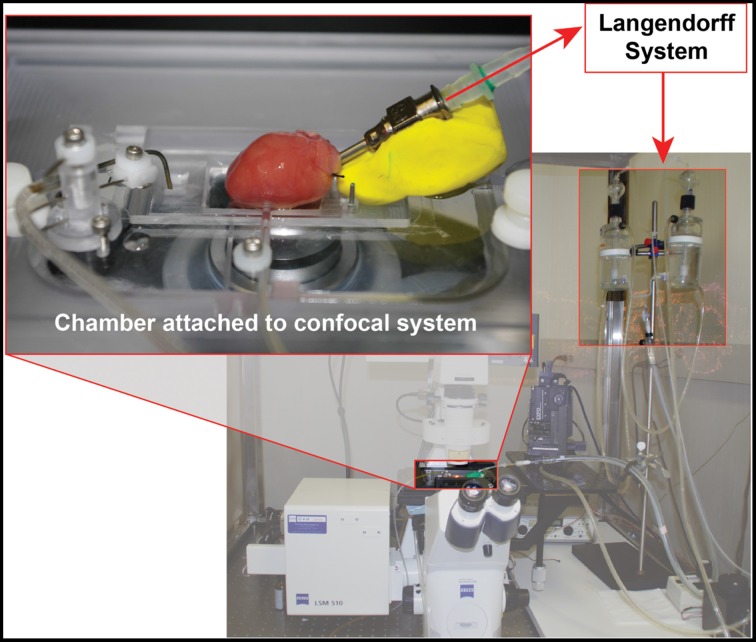
**Diagram of *in situ* confocal imaging system**.

In order to identifying subtle and comprehensive changes in T-tubule membrane structure of cardiomyocytes in their natural habitat, our group developed methods for *in situ* confocal imaging of T-tubules in intact hearts (Wei et al., 2010). First, we adapted the set-up of a laser scanning confocal microscope to include a Langendorff perfusion system as shown in Figure 1. The height of the perfusion apparatus is set at ~80 cm. The perfusate solution consists of normal Tyrode's solution with no Ca^2+^ (NaCl 137 mM, KCl 5.4 mM, HEPES 10 mM, Glucose 10 mM, MgCl_2_ 1 mM, NaH_2_PO_4_ 0.33 mM, pH adjusted to 7.4 with NaOH, oxygenated with 95% O_2_ and 5% CO_2_). The heart is perfused for ~30 min with perfusate containing membrane lipophilic marker MM 4-64 (AAT Bioquest, Inc) at a concentration of 5 μM. For T-tubule imaging, it is not necessary for the perfusate to be at physiological temperature, and thus most studies are performed at room temperature.

#### T-tubule image acquisition

**Figure 2:**
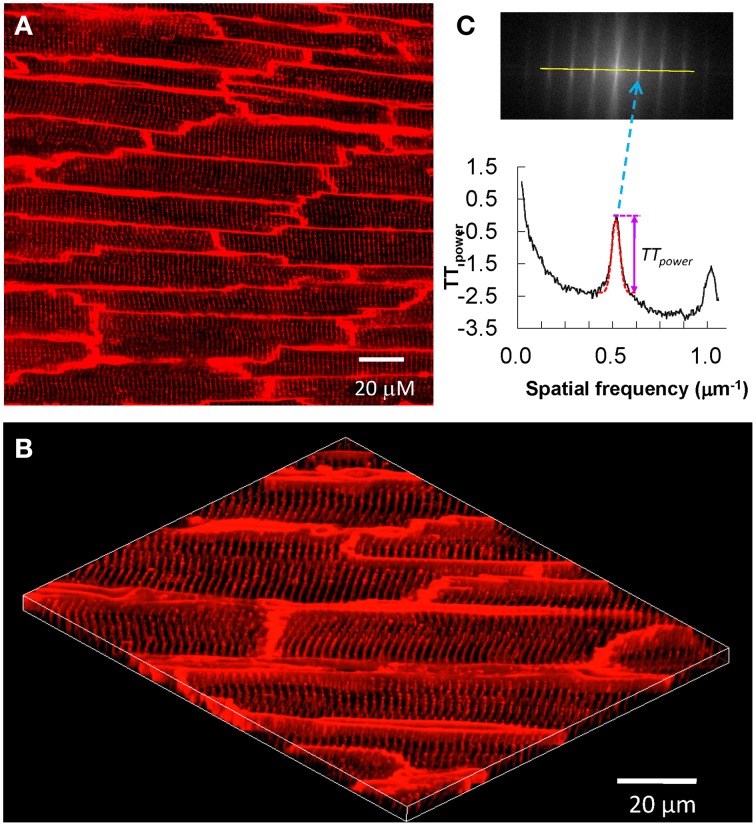
***In situ* confocal imaging of T-tubules on intact rat heart and T-tubule image analysis. (A)** A typical t-tubule image from the epicardium of Langendorff-perfused intact rat heart (control, sham-operated) loaded with lipophilic membrane indicator FM4-64. **(B)** The periodically organized T-tubules were viewed from a 3D reconstruction of 25 confocal stacks at 0.2 μm interval. Scale bar: 20 μm. **(C)** Power spectrum retrieved from a 2D Fourier transformation of T-tubule image characterized the power magnitude of the regular organization of T-tubule system. Data are adapted from Wei et al. (2010).

After T-tubule staining is completed, the heart is transferred to a chamber attached to a laser scanning confocal microscope, with the region of interested positioned to face the lens (Figure 1). For larger species (e.g., rat), sub-regions of the heart can be imaged, such as posterior or anterior LV. Specific imaging parameters are detailed as follows. The laser scanning confocal microscope is equipped with an oil immersion optical lens (40X or 63X, NA = 1.3). The laser excitation for MM 4-64 is 488, 543 or 561 nm, due to its broad excitation spectrum, whose power is comparable to that used for imaging of isolated myocytes. The optical pinhole was set to 1 airy disc. The maximum depth for high resolution imaging in our system is ~70 μm, which limits imaging to subepicardial myocytes. Practically, we choose the focus plane that yields the best image quality. With a 40X lens, each scanning frame (1024 × 1024 pixels) covers a 202 × 202 μm^2^ area, which contains on average 10–20 myocytes (Figure 2). Scanning sequentially along the surface of the heart allows for visualization of T-tubules in different subepicardial regions of an intact heart. For more thorough analyses, one can perform sequential imaging along the Z-axis at desired intervals in addition to planar (XY) scanning.

#### T-tubule image analysis

The most important characteristics of the myocyte T-tubule system is the highly organized, periodic pattern with regular spacing (~2 μm distance). Approaches to determine the integrity of the T-tubule system include measuring the density (area) (He et al., 2001; Heinzel et al., 2008; Lyon et al., 2009; Pavlovic et al., 2010; Kemi et al., 2011; Ibrahim et al., 2013) and regularity based on the power of the frequency spectrum of spatially repeated elements (TT_power_). (Song et al., 2006; Wei et al., 2010; Kemi et al., 2011; Wu et al., 2011; Swift et al., 2012; Ibrahim et al., 2013). However, caveats that are unique to each of these two methods limit data interpretation and quantitative comparison of images acquired from different studies. To overcome these limitations, we recently developed a program, AutoTT, which performs automated analysis of T-tubule images (Guo and Song, 2014). AutoTT is freely available to academic investigators. Features of AutoTT include (1) automated analysis of T-tubules; (2) image preprocessing to remove noise; (3) minimal influence of image brightness on data analysis (grayscale images are converted to binary black-and-white images); (4) enhanced comparison among images via analysis of topological architecture (sketches) rather than raw images and normalization of parameter(s). While AutoTT is optimized for analysis of single myocytes, a revised version allows for analysis of T-tubules in images of intact hearts. The original version, using images of single myocytes, removes cellular boundaries and analyzes the remaining intracellular structures. Analysis of images from intact hearts in the revised AutoTT program requires manual selection of intracellular structures, which can then be subjected to automated analysis of T-tubule density and regularity.

#### Advantages of *in situ* confocal T-tubules imaging

- Ability to image cardiac (ultra)structure in its native environment.
- Avoids artifacts associated with dissociation of cardiomyocytes.
- Subtle changes in T-tubule architecture can be visualized and quantitated.
- Ability to visualize T-tubules in multiple regions of the heart, including atrial, LV and RV epicardium.
- In larger species (e.g., rat), sub-regions of the heart can be imaged, such as posterior or anterior LV.
- For studies of infarcted hearts, it is relatively easier to image the infarct, border and remote zones.
- Technique can also be applied to image other features in intact hearts, such as Ca^2+^ handling and mitochondrial properties (reactive oxygen species, mitochondrial membrane potential, etc.).

#### Disadvantages of *in situ* confocal T-tubule imaging

- *In situ* T-tubule imaging is much more costly as compared to imaging in dissociated myocytes (i.e., requires a significantly larger amount of MM 4-64 per each experiment).
- Confocal microscope set-up must be adapted to incorporate Langendorff perfusion system.
- Imaging is restricted to subepicardial myocytes due to limitations in laser penetration; endocardium cannot be imaged.
- *In situ* imaging is not applicable for imaging subepicardium in large mammals (e.g., dog, pig or human samples, due to the thickness of the epicardium).

#### Two additional notes

- *Phototoxicity issue:* We do not expect greater phototoxicity in *in situ* imaging as compared to single cell imaging. This is because the laser power used for *in situ* imaging is highly comparable to that used for single cell imaging.
- *Temporal degradation:* This should not be a concern. First the experiments are performed at room temperature as used for isolated myocytes. For each heart, experiments can be done within 1–1.5 h (starting from excision of the heart from the animal). This length of time is even shorter than the approach of using isolated myocytes. Isolated cells are usually studied several hours (e.g., 4–5 h) after isolation.

### Novel findings using *in situ* confocal imaging of T-tubules

Studies using electron microscopy and laser scanning confocal imaging of fixed tissue samples or isolated cardiomyocytes provided the first evidence that T-tubules are altered in failing hearts (Maron et al., 1975; Schaper et al., 1991; Kostin et al., 1998; Kaprielian et al., 2000; He et al., 2001; Cannell et al., 2006; Louch et al., 2006; Heinzel et al., 2008; Lyon et al., 2009). However, it was unknown at the time whether pathological T-tubule remodeling only occurs in failing hearts, or if it starts earlier, e.g., in the compensated hypertrophy stage, and if so, how it evolves during the transition from hypertrophy to heart failure. Application of *in situ* T-tubule imaging has revealed that not only is T-tubule remodeling a mediator of heart failure, but T-tubules are critical for normal cardiac development and function. In this section, we review how *in situ* confocal imaging has provided key insights into the timing and regional differences in T-tubule integrity following cardiac stress and in developing hearts. In addition, we describe current efforts to prevent or reverse deleterious T-tubule remodeling.

#### T-tubule remodeling in animal models of heart failure

The first application of *in situ* confocal imaging of T-tubules was applied to a rat pressure overload model of cardiac stress (Wei et al., 2010). Following thoracic aortic banding, we utilized *in situ* T-tubule imaging to investigate the natural evolution of T-tubule remodeling over a spectrum of heart disease (compensated hypertrophy, early heart failure and advanced heart failure). We found that discrete local loss and global reorganization of the T-tubule system starts early in compensated hypertrophy in LV myocytes, prior to detectable LV dysfunction as assessed by echocardiography. At the onset, this process was manifested as subcellular T-tubule loss limited to discrete local regions in some LV myocytes, while the T-tubule structure in right ventricular (RV) myocytes was unaffected. However, with progression from compensated hypertrophy to early and late heart failure, T-tubule remodeling spreads from the LV to the RV. By the advanced stage of heart failure, the severity of T-tubule disruption in RV myocytes is equivalent to that seen in LV myocytes.

**Figure 3:**
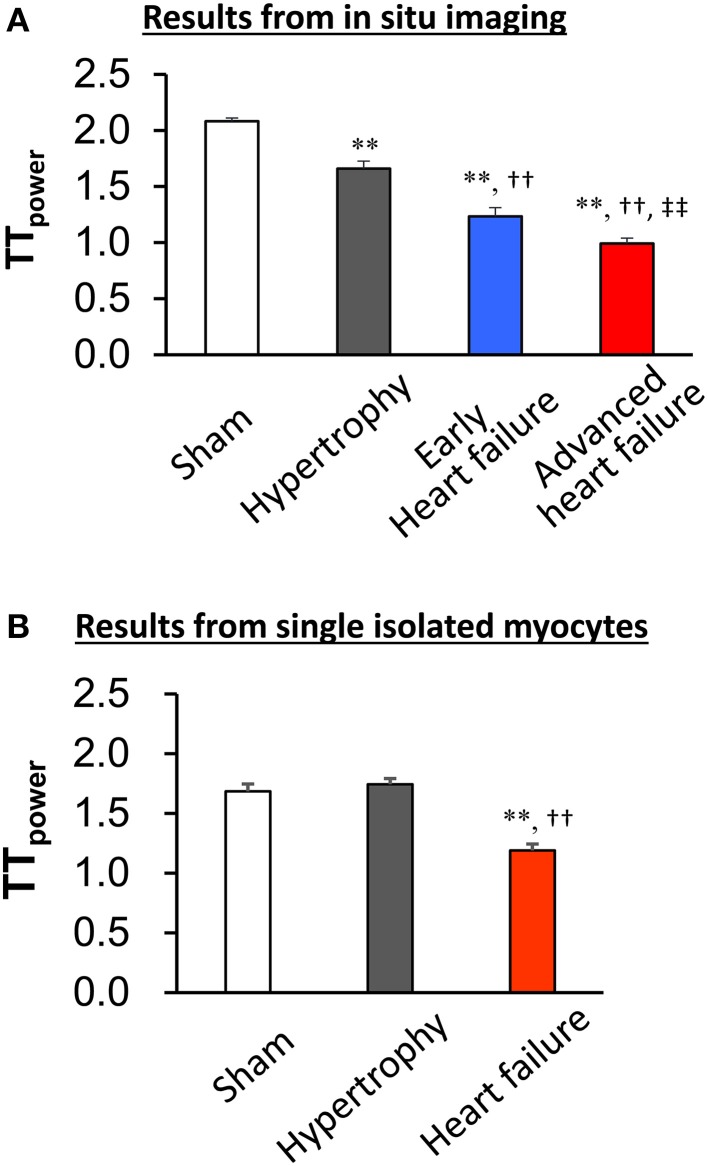
***In situ* imaging reveals T-tubule damage in both compensated and decompensated hypertrophy (A)**. By contrast, the TT_power_ in isolated myocytes from sham and hypertrophied TAB hearts is similar **(B)**, indicating that the isolation process results in damage to T-tubules. This damage prevents comparative quantitative analysis of T-tubule integrity following hypertrophy. ^**^P < 0.01 vs. Sham, ^††^*P* < 0.01 vs. Hypertrophy, ^‡‡^*P* < 0.01 vs. Early heart failure, *n* = 5–8.

This work provided the first evidence that T-tubule remodeling may constitute a key mechanism underlying the transition from compensated hypertrophy to heart failure. Use of *in situ* confocal T-tubule imaging also allows comparison of T-tubule integrity in different regions within the same heart, i.e., LV and RV myocytes. These data revealed that T-tubule damage is not a response to global neurohormonal changes, which would manifest as similar changes in LV and RV myocytes at each stage in heart failure progression. In addition, *in situ* imaging provides an opportunity to visualize and quantitate subtle changes in T-tubule architecture which may not be apparent in isolated myocytes. For example, in unpublished studies of isolated myocytes from pressure overload hypertrophied hearts, we did not observe changes in the T-tubule integrity as compared to sham control hearts (Figure 3). This is in contrast to our published findings that T-tubule remodeling is apparent in intact hearts in the compensated hypertrophy stage.

**Figure 4:**
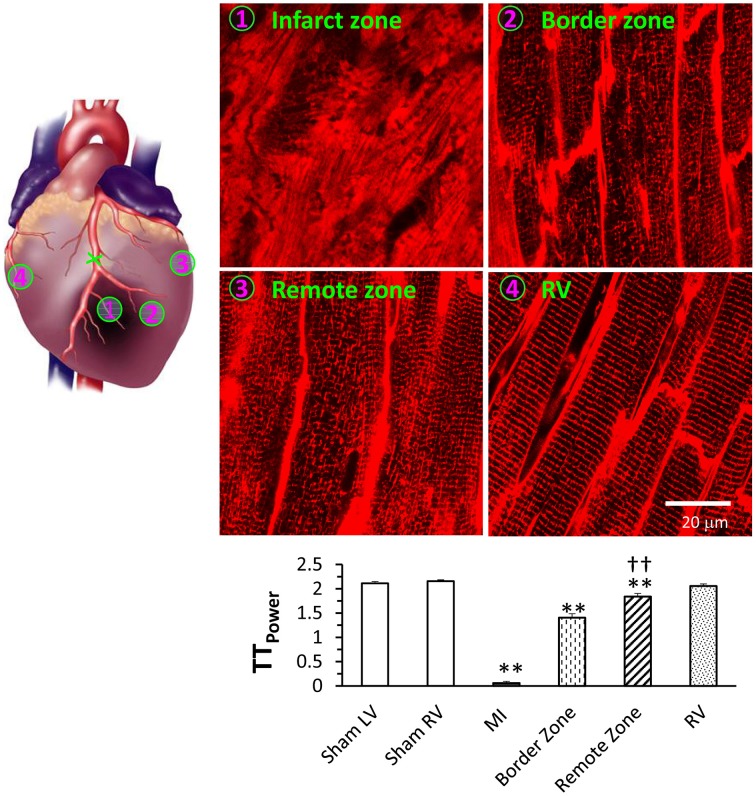
**Differential T-tubule remodeling in the border and remote zones relative to the infarct region as evidenced by *in situ* confocal imaging**. Images from different regions of the same heart can be captured and quantitated to compare the extent of T-tubule damage these various regions. Representative T-tubule images were acquired from regions as indicated. Bottom panel shows average data of TT_power_ from different regions. **P < 0.01 vs. sham LV, ^††^*P* < 0.01 vs. MI border zone, *P* < 0.01 among groups. Data are adapted from Chen et al. (2012a).

*In situ* confocal imaging has demonstrated that T-tubule remodeling is a common phenotype in several different models of heart failure, including rat models of pulmonary artery hypertension or systemic hypertension (Xie et al., 2012; Shah et al., 2014), mouse models of ischemic or pressure overload cardiomyopathy (Chen et al., 2012a; Guo et al., 2014), and mouse models with genetic modifications (Wu et al., 2011; Tao et al., 2012). Using a murine model of myocardial infarction, we have also investigated the severity of T-tubule remodeling in different regions relative to the infarct (Figure 4) (Chen et al., 2012a). *In situ* imaging demonstrated that myocardial infarction causes remarkable T-tubule remodeling near the infarction border zone (the area adjacent to the infarction), moderate remodeling in LV remote from the myocardial infarction (remote zone), and no damage to RV T-tubules. Quantitative analysis showed the TT_power_ is significantly different between the border and remote zones, suggesting that the damage to the border zone is more pronounced but the remote zone is also affected (Figure 4). Similar to the findings with the pressure overload model, where T-tubule remodeling began in LV myocytes and extended to RV myocytes with disease progression, these data using the MI model support the hypothesis that T-tubule damage is not due to defects in global neurohormonal system.

In addition to studies of T-tubule remodeling in diseased hearts, we also used *in situ* confocal imaging to determine the time course of T-tubule maturation in developing rodent hearts (Chen et al., 2013). In mice, T-tubules are absent in both left and right ventricular murine myocytes as late as postnatal day 8 and do not appear until day 10. T-tubules progressively increase between postnatal days 10 and 19, when it is similar to adult murine cardiomyocytes. Similar results were observed in rat hearts, though on a delayed time scale. Minimal T-tubules were apparent at postnatal day 13, significantly increased at day 15, and continued to gradually mature through day 23. These data are in line with previous reports using confocal imaging of isolated myocytes from murine and rat hearts (Ziman et al., 2010; Hamaguchi et al., 2013), though with *in situ* imaging we were able to demonstrate that T-tubule development in murine hearts begins as early as postnatal day 10. Studies with isolated myocytes only visualized a T-tubule network beginning at day 14 (Hamaguchi et al., 2013). The development of the T-tubule system in larger animals is likely later as compared to rodents (Haddock et al., 1999). Interestingly, our imaging analysis also revealed that the density of transverse but not longitudinal tubules gradually increases during development. This study highlights the utility of *in situ* confocal imaging to visualize T-tubule organization in cardiomyocytes from very young mice, which are difficult to isolate and frequently lose their native morphology after dissociation.

#### Prevention of T-tubule remodeling or restoration of T-tubule integrity

Building on *in situ* imaging results that establish T-tubule remodeling as a key mediator of heart failure, we have also asked if strategies to restore cardiac function prevent or reverse T-tubule damage. In one study, we used a murine model of myocardial infarction to examine how β-adrenergic receptor blockade impacts T-tubule ultrastructure. (Chen et al., 2012a) β-adrenergic receptor blockers metoprolol and carvedilol are widely prescribed to improve LV function after infarction, yet the full mechanism of drug action is not clear. Mice were administered metoprolol or carvedilol 3 days after surgery, and then T-tubule integrity quantitated by *in situ* confocal imaging at 5 weeks post infarction. Our study demonstrates that both metoprolol and carvedilol protect against T-tubule remodeling in both the remote and border zones. These data provide evidence that the beneficial effect provided by β-adrenergic receptor antagonist includes protection against T-tubule remodeling post infarction. In a parallel study, we found that sildenafil, a phosphodiesterase type 5 inhibitor that was recently approved for treatment of pulmonary artery hypertension, prevents and partially reverses ultrastructural remodeling of failing right ventricular myocytes (Xie et al., 2012).

T-tubule imaging also provided key mechanistic insights into how the increase in microtubule density upon cardiac stress is linked to LV contractile dysfunction. Microtubules are ubiquitous cytoskeletal fibers formed by polymerization of α- and β-tubulin dimers. Excessive microtubule polymerization (“densification”) is a common observation in multiple animal models of cardiac disease and is associated with adverse cardiac phenotypes in humans. (Tsutsui et al., 1993, 1994; Heling et al., 2000; Zile et al., 2001) Our recently reported study showed that microtubule depolymerization with colchicine profoundly attenuates T-tubule impairment and improves cardiac function following cardiac stress. Our study further suggests that microtubule densification mediates junctophilin-2 mis-trafficking and thus abnormal localization, causing T-tubule alterations and contractile dysfunction under pressure overload stress (Zhang et al., 2014).

In our studies of T-tubule remodeling in multiple models of heart failure, we consistently observed a loss in the structure protein junctophilin-2, which is implicated in the formation of T-tubule/SR junctions and thereby E–C coupling. For example, we and others have observed a significant loss in the expression of junctophilin-2 in multiple different models of heart failure. (Minamisawa et al., 2004; Landstrom et al., 2007; Xu et al., 2007; Wei et al., 2010; Chen et al., 2012a; Wu et al., 2012; Xie et al., 2012). Furthermore, deficiency of junctophilin-2 prevents maturation of the T-tubule system in developing murine hearts (Chen et al., 2013). In light of the emerging importance of junctophilin-2 in cardiac excitation–contraction coupling and T-tubule remodeling in health and disease, we generated transgenic mice with inducible cardiac specific overexpression of junctophilin-2 protein. While mice are normal at baseline, constitutive transgenic junctophilin-2 overexpression protects against heart failure by maintaining T-tubule structural integrity following cardiac stress (Guo et al., 2014). The design of this model will allow in the future to determine whether augmenting junctophilin-2 expression is a strategy to reverse T-tubule damage.

**Figure 5:**
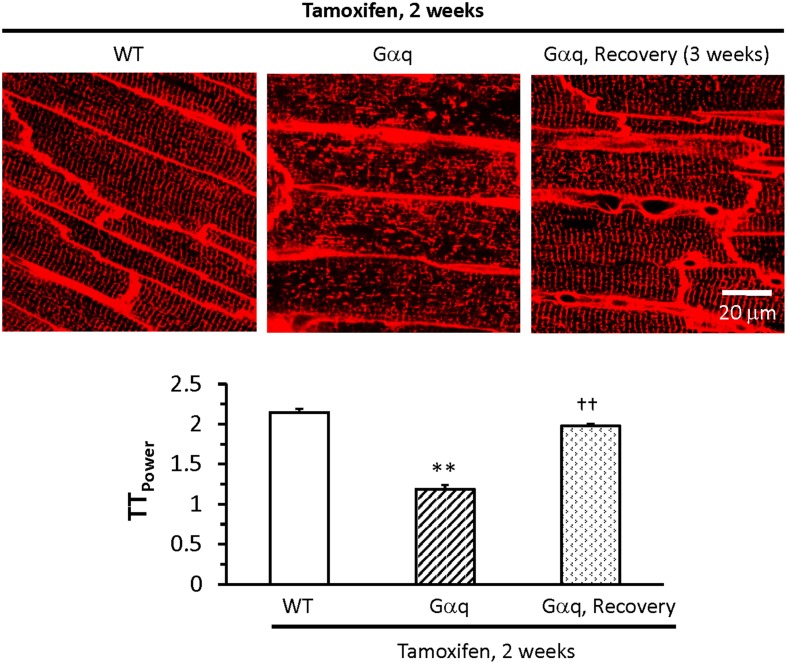
**T-tubule damage is reversible**. Top panel displays *in situ* images of T-tubules from the LV of wildtype (WT – left) and Gαq mice (center) treated with tamoxifen for 2 weeks, and Gαq mice treated with tamoxifen for 2 weeks following by tamoxifen withdrawal for 3 weeks (recovery – right). Bottom panel shows quantitative assessment of T-tubule organization by power spectrum analysis (TT_power_). T-tubule integrity in the Gαq recovery group was almost restored to normal levels. **P < 0.01 vs. WT, ^††^*P* < 0.01 vs Gαq mice treated with tamoxifen for 2 weeks. Data are adapted from Wu et al. (2014).

The most compelling evidence that T-tubule remodeling can be reversed was obtained through studies of a reversible model of heart failure via inducible transgenic expression of the G protein coupled receptor Gα_q_ (Wu et al., 2014). Upon Gα_q_ activation, the transgenic mice exhibit heart failure which is reversed by cessation of Gα_q_ activation. More importantly, we found transgenic Gα_q_ expression causes a severe disruption of the T-tubule network and loss of junctophilin-2 expression in transgenic Gα_q_ hearts, which could also be reversed by turning off Gα_q_ (Figure 5). In addition, treatment with a calpain inhibitor at the time of Gα_q_ induction prevents T-tubule remodeling and junctophilin-2 loss. The proposed mechanism is that cardiac stress results in calpain-mediated degradation of junctophilin-2, T-tubule remodeling and E–C coupling dysfunction. Strategies to prevent the loss of junctophilin-2 may therefore represent an approach to restore cardiac function after initial T-tubule damage.

## Conclusions

The power of *in situ* confocal imaging to provide novel insights into the cardiac ultrastructure and function in intact hearts is undeniable. However, this methodology has not achieved wide-spread application because of technical considerations and experience. For example, *in situ* T-tubule imaging requires a dedicated confocal microscope with a Langendorff perfusion system, which is not practical for laboratories that rely on core facilities for microscopy needs. In addition, a certain degree of technical skill is required to acquire high quality images. The applicability of *in situ* confocal imaging is not restricted to T-tubules. Indeed, it can be used to image other parameters critical for cardiac function, such as action potential (Bu et al., 2009), Ca^2+^ dynamics (Kaneko et al., 2000; Rubart et al., 2003; Aistrup et al., 2006, 2009; Fujiwara et al., 2008; Chen et al., 2012b; Zhang et al., 2013), mitochondrial structure (Wei et al., 2010; see Supplemental data of this reference) and function (Zhang et al., 2013) and studies of free radicals (Wang et al., 2008), etc.

### Conflict of interest statement

The authors declare that the research was conducted in the absence of any commercial or financial relationships that could be construed as a potential conflict of interest.

## References

[B1] Aistrup G. L. Kelly J. E. Kapur S. Kowalczyk M. Sysman-Wolpin I. Kadish A. H. (2006). Pacing-induced heterogeneities in intracellular Ca2+ signaling, cardiac alternans, and ventricular arrhythmias in intact rat heart. Circ. Res. 99, e65–73 10.1161/01.RES.0000244087.36230.bf 16960102

[B2] Aistrup G. L. Shiferaw Y. Kapur S. Kadish A. H. Wasserstrom J. A. (2009). Mechanisms underlying the formation and dynamics of subcellular calcium alternans in the intact rat heart. Circ. Res. 104, 639–649. 10.1161/CIRCRESAHA.108.181909 19150887

[B3] Ayettey A. S. Navaratnam V. (1978). The T-tubule system in the specialized and general myocardium of the rat. J. Anat. 127, 125–140. 701190 PMC1235649

[B4] Brette F. Orchard C. (2003). T-tubule function in mammalian cardiac myocytes. Circ. Res. 92, 1182–1192. 10.1161/01.RES.0000074908.17214.FD 12805236

[B5] Bu G. Adams H. Berbari E. J. Rubart M. (2009). Uniform action potential repolarization within the sarcolemma of *in situ* ventricular cardiomyocytes. Biophys. J. 96, 2532–2546. 10.1016/j.bpj.2008.12.3896 19289075 PMC2907679

[B6] Cannell M. B. Crossman D. J. Soeller C. (2006). Effect of changes in action potential spike configuration, junctional sarcoplasmic reticulum micro-architecture and altered t-tubule structure in human heart failure. J. Muscle Res. Cell Motil. 27, 297–306 10.1007/s10974-006-9089-y 16897575

[B7] Chazotte B. (2011). Labeling membrane glycoproteins or glycolipids with fluorescent wheat germ agglutinin. Cold Spring Harb. Protoc. 2011:pdb prot5623. 10.1101/pdb.prot5623 21536757

[B8] Chen B. Guo A. Gao Z. Wei S. Xie Y. P. Chen S. R. (2012b). *In situ* confocal imaging in intact heart reveals stress-induced Ca(2+) release variability in a murine catecholaminergic polymorphic ventricular tachycardia model of type 2 ryanodine receptor(R4496C+/−) mutation. Circ. Arrhythm. Electrophysiol. 5, 841–849 10.1161/CIRCEP.111.969733 22722659 PMC3421047

[B9] Chen B. Guo A. Zhang C. Chen R. Zhu Y. Hong J. (2013). Critical roles of junctophilin-2 in T-tubule and excitation-contraction coupling maturation during postnatal development. Cardiovasc. Res. 100, 54–62 10.1093/cvr/cvt180 23860812 PMC3778961

[B10] Chen B. Li Y. Jiang S. Xie Y. P. Guo A. Kutschke W. (2012a). beta-Adrenergic receptor antagonists ameliorate myocyte T-tubule remodeling following myocardial infarction. FASEB J. 26, 2531–2537 10.1096/fj.11-199505 22375019 PMC3360148

[B11] Crossman D. J. Ruygrok P. N. Soeller C. Cannell M. B. (2011). Changes in the organization of excitation-contraction coupling structures in failing human heart. PLoS ONE 6:e17901. 10.1371/journal.pone.0017901 21408028 PMC3052389

[B12] Di Maio A. Ter Keurs H. E. Franzini-Armstrong C. (2007). T-tubule profiles in Purkinje fibres of mammalian myocardium. J. Muscle Res. Cell Motil. 28, 115–121. 10.1007/s10974-007-9109-6 17572852

[B13] Dibb K. M. Clarke J. D. Horn M. A. Richards M. A. Graham H. K. Eisner D. A.. (2009). Characterization of an extensive transverse tubular network in sheep atrial myocytes and its depletion in heart failure. Circ. Heart Fail. 2, 482–489. 10.1161/CIRCHEARTFAILURE.109.852228 19808379

[B14] Fawcett D. W. McNutt N. S. (1969). The ultrastructure of the cat myocardium. I. Ventricular papillary muscle. J. Cell Biol. 42, 1–45. 10.1083/jcb.42.1.1 4891913 PMC2107571

[B15] Fujiwara K. Tanaka H. Mani H. Nakagami T. Takamatsu T. (2008). Burst emergence of intracellular Ca2+ waves evokes arrhythmogenic oscillatory depolarization via the Na+-Ca2+ exchanger: simultaneous confocal recording of membrane potential and intracellular Ca2+ in the heart. Circ Res. 103, 509–518. 10.1161/CIRCRESAHA.108.176677 18635824

[B70] Guo A. Song L. S. (2014). AutoTT: automated detection and analysis of T-tubule architecture in cardiomyocytes. Biophys. J. 106, 2729–2736. 10.1016/j.bpj.2014.05.013 24940790 PMC4070273

[B16] Guo A. Zhang C. Wei S. Chen B. Song L. S. (2013). Emerging mechanisms of T-tubule remodelling in heart failure. Cardiovasc. Res. 98, 204–215. 10.1093/cvr/cvt020 23393229 PMC3697065

[B17] Guo A. Zhang X. Iyer V. R. Chen B. Zhang C. Kutschke W. J. (2014). Overexpression of junctophilin-2 does not enhance baseline function but attenuates heart failure development after cardiac stress. Proc. Natl. Acad. Sci. U.S.A. 111, 12240–12245 10.1073/pnas.1412729111 25092313 PMC4143026

[B18] Haddock P. S. Coetzee W. A. Cho E. Porter L. Katoh H. Bers D. M.. (1999). Subcellular [Ca2+]i gradients during excitation-contraction coupling in newborn rabbit ventricular myocytes. Circ. Res. 85, 415–427. 10.1161/01.RES.85.5.415 10473671

[B19] Hamaguchi S. Kawakami Y. Honda Y. Nemoto K. Sano A. Namekata I.. (2013). Developmental changes in excitation-contraction mechanisms of the mouse ventricular myocardium as revealed by functional and confocal imaging analyses. J. Pharmacol. Sci. 123, 167–175. 10.1254/jphs.13099FP 24096881

[B20] Hayashi T. Martone M. E. Yu Z. Thor A. Doi M. Holst M. J.. (2009). Three-dimensional electron microscopy reveals new details of membrane systems for Ca2+ signaling in the heart. J. Cell Sci. 122, 1005–1013. 10.1242/jcs.028175 19295127 PMC2720931

[B21] He J. Conklin M. W. Foell J. D. Wolff M. R. Haworth R. A. Coronado R.. (2001). Reduction in density of transverse tubules and L-type Ca(2+) channels in canine tachycardia-induced heart failure. Cardiovasc. Res. 49, 298–307. 10.1016/S0008-6363(00)00256-X 11164840

[B22] Heinzel F. R. Bito V. Biesmans L. Wu M. Detre E. von Wegner F.. (2008). Remodeling of T-tubules and reduced synchrony of Ca2+ release in myocytes from chronically ischemic myocardium. Circ. Res. 102, 338–346. 10.1161/CIRCRESAHA.107.160085 18079411

[B23] Heling A. Zimmermann R. Kostin S. Maeno Y. Hein S. Devaux B.. (2000). Increased expression of cytoskeletal, linkage, and extracellular proteins in failing human myocardium. Circ. Res. 86, 846–853. 10.1161/01.RES.86.8.846 10785506

[B24] Ibrahim M. Al Masri A. Navaratnarajah M. Siedlecka U. Soppa G. K. Moshkov A. (2010). Prolonged mechanical unloading affects cardiomyocyte excitation-contraction coupling, transverse-tubule structure, and the cell surface. FASEB J. 24, 3321–3329 10.1096/fj.10-156638 20430793 PMC2923356

[B25] Ibrahim M. Gorelik J. Yacoub M. H. Terracciano C. M. (2011). The structure and function of cardiac t-tubules in health and disease. Proc. Biol. Sci. 278, 2714–2723. 10.1098/rspb.2011.0624 21697171 PMC3145195

[B26] Ibrahim M. Siedlecka U. Buyandelger B. Harada M. Rao C. Moshkov A.. (2013). A critical role for Telethonin in regulating t-tubule structure and function in the mammalian heart. Hum. Mol. Genet. 22, 372–383. 10.1093/hmg/dds434 23100327 PMC3526164

[B27] Jayasinghe I. D. Cannell M. B. Soeller C. (2009). Organization of ryanodine receptors, transverse tubules, and sodium-calcium exchanger in rat myocytes. Biophys. J. 97, 2664–2673. 10.1016/j.bpj.2009.08.036 19917219 PMC2776253

[B28] Kaneko T. Tanaka H. Oyamada M. Kawata S. Takamatsu T. (2000). Three distinct types of Ca(2+) waves in Langendorff-perfused rat heart revealed by real-time confocal microscopy. Circ. Res. 86, 1093–1099. 10.1161/01.RES.86.10.1093 10827140

[B29] Kaprielian R. R. Stevenson S. Rothery S. M. Cullen M. J. Severs N. J. (2000). Distinct patterns of dystrophin organization in myocyte sarcolemma and transverse tubules of normal and diseased human myocardium. Circulation 101, 2586–2594. 10.1161/01.CIR.101.22.2586 10840009

[B30] Kemi O. J. Hoydal M. A. Macquaide N. Haram P. M. Koch L. G. Britton S. L.. (2011). The effect of exercise training on transverse tubules in normal, remodeled, and reverse remodeled hearts. J. Cell Physiol. 226, 2235–2243. 10.1002/jcp.22559 21660947 PMC3096693

[B31] Kostin S. Scholz D. Shimada T. Maeno Y. Mollnau H. Hein S.. (1998). The internal and external protein scaffold of the T-tubular system in cardiomyocytes. Cell Tissue Res. 294, 449–460. 10.1007/s004410051196 9799462

[B32] Landstrom A. P. Weisleder N. Batalden K. B. Bos J. M. Tester D. J. Ommen S. R.. (2007). Mutations in JPH2-encoded junctophilin-2 associated with hypertrophic cardiomyopathy in humans. J. Mol. Cell Cardiol. 42, 1026–1035. 10.1016/j.yjmcc.2007.04.006 17509612 PMC4318564

[B33] Louch W. E. Mork H. K. Sexton J. Stromme T. A. Laake P. Sjaastad I. (2006). T-tubule disorganization and reduced synchrony of Ca2+ release in murine cardiomyocytes following myocardial infarction. J. Physiol. 574, 519–533 10.1113/jphysiol.2006.107227 16709642 PMC1817777

[B34] Lyon A. R. MacLeod K. T. Zhang Y. Garcia E. Kanda G. K. Lab M. J.. (2009). Loss of T-tubules and other changes to surface topography in ventricular myocytes from failing human and rat heart. Proc. Natl. Acad. Sci. U.S.A. 106, 6854–6859. 10.1073/pnas.0809777106 19342485 PMC2672472

[B35] Maron B. J. Ferrans V. J. Roberts W. C. (1975). Ultrastructural features of degenerated cardiac muscle cells in patients with cardiac hypertrophy. Am. J. Pathol. 79, 387–434. 124533 PMC1912738

[B36] Minamisawa S. Oshikawa J. Takeshima H. Hoshijima M. Wang Y. Chien K. R.. (2004). Junctophilin type 2 is associated with caveolin-3 and is down-regulated in the hypertrophic and dilated cardiomyopathies. Biochem. Biophys. Res. Commun. 325, 852–856. 10.1016/j.bbrc.2004.10.107 15541368

[B37] Nelson D. A. Benson E. S. (1963). On the structural continuities of the transverse tubular system of rabbit and human myocardial cells. J. Cell Biol. 16, 297–313. 10.1083/jcb.16.2.297 13938025 PMC2106241

[B38] Ohler A. Weisser-Thomas J. Piacentino V. Houser S. R. Tomaselli G. F. O'Rourke B. (2009). Two-photon laser scanning microscopy of the transverse-axial tubule system in ventricular cardiomyocytes from failing and non-failing human hearts. Cardiol. Res. Pract. 2009:802373 10.4061/2009/802373 20224636 PMC2833295

[B39] Pavlovic D. McLatchie L. M. Shattock M. J. (2010). The rate of loss of T-tubules in cultured adult ventricular myocytes is species dependent. Exp. Physiol. 95, 518–527. 10.1113/expphysiol.2009.052126 20061354

[B40] Richards M. A. Clarke J. D. Saravanan P. Voigt N. Dobrev D. Eisner D. A. (2011). Transverse tubules are a common feature in large mammalian atrial myocytes including human. Am. J. Physiol. Heart Circ. Physiol. 301, H1996–H2005 10.1152/ajpheart.00284.2011 21841013 PMC3213978

[B41] Rostgaard J. Behnke O. (1965). Fine structural localization of adenine nucleoside phosphatase activity in the sarcoplasmic reticulum and the T system of rat myocardium. J. Ultrastruct. Res. 12, 579–591. 10.1016/S0022-5320(65)80049-1 4284093

[B42] Rubart M. Wang E. Dunn K. W. Field L. J. (2003). Two-photon molecular excitation imaging of Ca2+ transients in Langendorff-perfused mouse hearts. Am. J. Physiol. Cell Physiol. 284, C1654–C1668. 10.1152/ajpcell.00469.2002 12584115

[B43] Sachse F. B. Torres N. S. Savio-Galimberti E. Aiba T. Kass D. A. Tomaselli G. F. (2012). Subcellular structures and function of myocytes impaired during heart failure are restored by cardiac resynchronization therapy. Circ. Res. 110, 588–597 10.1161/CIRCRESAHA.111.257428 22253411 PMC3299196

[B44] Savio-Galimberti E. Frank J. Inoue M. Goldhaber J. I. Cannell M. B. Bridge J. H. (2008). Novel features of the rabbit transverse tubular system revealed by quantitative analysis of three-dimensional reconstructions from confocal images. Biophys. J. 95, 2053–2062 10.1529/biophysj.108.130617 18487298 PMC2483780

[B45] Schaper J. Froede R. Hein S. Buck A. Hashizume H. Speiser B.. (1991). Impairment of the myocardial ultrastructure and changes of the cytoskeleton in dilated cardiomyopathy. Circulation 83, 504–514. 10.1161/01.CIR.83.2.504 1991369

[B46] Shah S. J. Aistrup G. L. Gupta D. K. O'Toole M. J. Nahhas A. F. Schuster D.. (2014). Ultrastructural and cellular basis for the development of abnormal myocardial mechanics during the transition from hypertension to heart failure. Am. J. Phys. Heart Circ. Physiol. 306, H88–H100. 10.1152/ajpheart.00642.2013 24186100 PMC3920157

[B47] Simpson F. O. (1965). The transverse tubular system in mammalian myocardial cells. Am. J. Anat. 117, 1–17. 10.1002/aja.1001170102 14345833

[B48] Soeller C. Cannell M. B. (1999). Examination of the transverse tubular system in living cardiac rat myocytes by 2-photon microscopy and digital image-processing techniques. Circ. Res. 84, 266–275. 10.1161/01.RES.84.3.266 10024300

[B49] Song L. S. Sobie E. A. McCulle S. Lederer W. J. Balke C. W. Cheng H. (2006). Orphaned ryanodine receptors in the failing heart. Proc. Natl. Acad. Sci. U.S.A. 103, 4305–4310. 10.1073/pnas.0509324103 16537526 PMC1449688

[B50] Stern M. D. (1992). Theory of excitation-contraction coupling in cardiac muscle. Biophys. J. 63, 497–517. 10.1016/S0006-3495(92)81615-6 1330031 PMC1262173

[B51] Stolen T. O. Hoydal M. A. Kemi O. J. Catalucci D. Ceci M. Aasum E.. (2009). Interval training normalizes cardiomyocyte function, diastolic Ca2+ control, and SR Ca2+ release synchronicity in a mouse model of diabetic cardiomyopathy. Circ. Res. 105, 527–536. 10.1161/CIRCRESAHA.109.199810 19679837

[B52] Sun X. H. Protasi F. Takahashi M. Takeshima H. Ferguson D. G. Franzini-Armstrong C. (1995). Molecular architecture of membranes involved in excitation-contraction coupling of cardiac muscle. J. Cell Biol. 129, 659–671. 10.1083/jcb.129.3.659 7730402 PMC2120446

[B53] Swift F. Franzini-Armstrong C. Oyehaug L. Enger U. H. Andersson K. B. Christensen G.. (2012). Extreme sarcoplasmic reticulum volume loss and compensatory T-tubule remodeling after Serca2 knockout. Proc. Natl. Acad. Sci. U.S.A. 109, 3997–4001. 10.1073/pnas.1120172109 22355118 PMC3309775

[B54] Tao W. Shi J. Dorn G. W. II Wei L. Rubart M. (2012). Spatial variability in T-tubule and electrical remodeling of left ventricular epicardium in mouse hearts with transgenic Galphaq overexpression-induced pathological hypertrophy. J. Mol. Cell Cardiol. 53, 409–419 10.1016/j.yjmcc.2012.06.006 22728217 PMC3574572

[B55] Tsutsui H. Ishihara K. Cooper G. (1993). Cytoskeletal role in the contractile dysfunction of hypertrophied myocardium. Science 260, 682–687. 10.1126/science.8097594 8097594

[B56] Tsutsui H. Tagawa H. Kent R. L. McCollam P. L. Ishihara K. Nagatsu M.. (1994). Role of microtubules in contractile dysfunction of hypertrophied cardiocytes. Circulation 90, 533–555. 10.1161/01.CIR.90.1.533 8026043

[B57] Wagner E. Lauterbach M. A. Kohl T. Westphal V. Williams G. S. Steinbrecher J. H.. (2012). Stimulated emission depletion live-cell super-resolution imaging shows proliferative remodeling of T-tubule membrane structures after myocardial infarction. Circ. Res. 111, 402–414. 10.1161/CIRCRESAHA.112.274530 22723297 PMC4219578

[B58] Wang S. Q. Song L. S. Lakatta E. G. Cheng H. (2001). Ca2+ signalling between single L-type Ca2+ channels and ryanodine receptors in heart cells. Nature 410, 592–596. 10.1038/35069083 11279498

[B59] Wang W. Fang H. Groom L. Cheng A. Zhang W. Liu J.. (2008). Superoxide flashes in single mitochondria. Cell 134, 279–290. 10.1016/j.cell.2008.06.017 18662543 PMC2547996

[B60] Wei S. Guo A. Chen B. Kutschke W. Xie Y. P. Zimmerman K.. (2010). T-tubule remodeling during transition from hypertrophy to heart failure. Circ. Res. 107, 520–531. 10.1161/CIRCRESAHA.109.212324 20576937 PMC2927862

[B61] Wu C. Y. Chen B. Jiang Y. P. Jia Z. Martin D. W. Liu S.. (2014). Calpain-dependent cleavage of junctophilin-2 and T-tubule remodeling in a mouse model of reversible heart failure. J. Am. Heart Assoc. 3:e000527. 10.1161/JAHA.113.000527 24958777 PMC4309042

[B62] Wu C. Y. Jia Z. Wang W. Ballou L. M. Jiang Y. P. Chen B.. (2011). PI3Ks maintain the structural integrity of T-tubules in cardiac myocytes. PLoS ONE 6:e24404. 10.1371/journal.pone.0024404 21912691 PMC3166327

[B63] Wu H. D. Xu M. Li R. C. Guo L. Lai Y. S. Xu S. M. (2012). Ultrastructural remodelling of Ca2+ signalling apparatus in failing heart cells. Cardiovasc. Res. 95, 430–438 10.1093/cvr/cvs195 22707157 PMC3422078

[B64] Xie Y. P. Chen B. Sanders P. Guo A. Li Y. Zimmerman K. (2012). Sildenafil prevents and reverses transverse-tubule remodeling and Ca(2+) handling dysfunction in right ventricle failure induced by pulmonary artery hypertension. Hypertension 59, 355–362 10.1161/HYPERTENSIONAHA.111.180968 22203744 PMC3266850

[B65] Xu M. Zhou P. Xu S. M. Liu Y. Feng X. Bai S. H.. (2007). Intermolecular failure of L-type Ca2+ channel and ryanodine receptor signaling in hypertrophy. PLoS Biol. 5:e21. 10.1371/journal.pbio.0050021 17214508 PMC1764437

[B66] Zhang C. Chen B. Guo A. Zhu Y. Miller J. D. Gao S. (2014). Microtubule-mediated defects in junctophilin-2 trafficking contribute to myocyte transverse-tubule remodeling and Ca2+ handling dysfunction in heart failure. Circulation 129, 1742–1750 10.1161/CIRCULATIONAHA.113.008452 24519927 PMC4006305

[B67] Zhang H. Shang W. Zhang X. Gu J. Wang X. Zheng M.. (2013). Beta-adrenergic-stimulated L-type channel Ca(2)+ entry mediates hypoxic Ca(2)+ overload in intact heart. J. Mol. Cell. Cardiol. 65, 51–58. 10.1016/j.yjmcc.2013.09.002 24041537

[B68] Zile M. R. Green G. R. Schuyler G. T. Aurigemma G. P. Miller D. C. Cooper G. (2001). Cardiocyte cytoskeleton in patients with left ventricular pressure overload hypertrophy. J. Am. Coll. Cardiol. 37, 1080–1084 10.1016/S0735-1097(00)01207-9 11263612

[B69] Ziman A. P. Gomez-Viquez N. L. Bloch R. J. Lederer W. J. (2010). Excitation-contraction coupling changes during postnatal cardiac development. J. Mol. Cell. Cardiol. 48, 379–386 10.1016/j.yjmcc.2009.09.016 19818794 PMC3097073

